# Effects of a Lumbar Support Cushion in a Backpack on Postural Alignment and Skeletal Muscle Mechanical Properties: A Repeated-Measures Trial in Healthy Adults

**DOI:** 10.3390/jcm15207900

**Published:** 2026-10-13

**Authors:** Ju-Yeon Jung, Jin-Hwa Jung, Hwi-Young Cho, Ji-Eun Baek, Hye Rim Suh

**Affiliations:** 1 Institute of Human Convergence Health Science, Gachon University, Incheon 21936, Republic of Korea; 9955me@gachon.ac.kr; 2 Department of Occupational Therapy, Semyung University, Jecheon 27136, Republic of Korea; otsalt@semyung.ac.kr; 3 Department of Physical Therapy, College of Health Science, Gachon University, Incheon 21936, Republic of Korea; 4 Department of Physical Therapy, Division of Health Science, Baekseok University, Cheonan 31065, Republic of Korea

**Keywords:** backpack, detachable lumbar support cushion, postural alignment, muscle tone, muscle stiffness

## Abstract

**Background/Objectives:** Backpacks are widely used for carrying loads; however, posteriorly positioned loads can shift the body’s center of mass, promote forward trunk and head compensation, and increase neck and shoulder muscle loading. This study examined the effects of a detachable lumbar support cushion at loads equivalent to 10%, 15%, and 20% of the body weight. **Methods:** Twenty-eight healthy adult men completed repeated-measures trials of backpacks without or with lumbar support cushions. The craniovertebral angle (CVA), forward trunk lean (FTL), pelvic tilt angle (PTA), upper trapezius, sternocleidomastoid (SCM) tone, and stiffness were assessed. The trial registration was completed by the Clinical Research Information Service of the Republic of Korea (KCT0012211). **Results:** Most variables changed significantly as the load increased, with the largest changes occurring up to 15% and limited additional changes between 15% and 20%. The cushion did not significantly affect CVA or FTL but increased PTA and reduced SCM tone. **Conclusions:** A detachable lumbar support cushion may modify the PTA and was associated with lower SCM tone during backpack carriage.

## 1. Introduction

Backpacks are widely used by diverse populations ranging from school-aged children to university students and office workers. They allow the upper limbs to remain free, while providing a convenient means for carrying personal items. However, excessive loads and improper use may increase physical demands, alter posture, and contribute to ergonomic problems. Backpack carriage increases the mechanical loading on the lower back and upper trunk and may be associated with fatigue, low back pain, and other musculoskeletal symptoms [1,2].

The backpack load is an external factor that can alter lumbar lordosis and cervical curvature [3,4]. Backpack carriages have been shown to immediately reduce sagittal spinal curvature, particularly lumbar lordosis, during standing and may also impair repositioning ability [3]. In adults, carrying loads equivalent to 5–20% of body weight has repeatedly been associated with reductions in lumbar lordosis and upper thoracic kyphosis [5,6]. These changes in sagittal alignment may compromise spinal shock absorption and load distribution, thus highlighting the need for strategies to help preserve lumbar lordosis during backpack use.

Various backpack designs and carrying strategies have been proposed to reduce the biomechanical demands associated with carrying backpacks. Among these approaches, lumbar support may influence the interaction between the backpack and the lumbopelvic region, thereby modifying postural compensation during load carriage. Backpacks incorporating lumbar support can influence lumbar lordosis and spinal alignment [7]. However, most existing lumbar support systems are integrated into a backpack, which requires the use of a specific backpack design and thus limits their practical applicability. Therefore, a detachable lumbar support cushion, which can be applied to conventional backpacks, may provide a more flexible approach. Nevertheless, it remains unclear how a detachable lumbar support cushion influences the postural alignment and mechanical properties of the neck and shoulder muscles across different backpack loads in healthy young adults. Therefore, the present study investigated the effects of backpack load and a detachable lumbar support cushion on the postural alignment and mechanical properties of the upper trapezius (UT) and sternocleidomastoid (SCM) muscles in healthy young adults.

We hypothesized that increasing the backpack load would progressively alter postural alignment and increase the tone and stiffness of the UT and SCM muscles and that the use of a detachable lumbar support cushion would modify these load-related postural responses and attenuate increases in the mechanical load on neck and shoulder muscles compared with backpack carriage without the cushion.

Therefore, this study developed a detachable lumbar support cushion that can be used with a conventional backpack and investigated the changes in postural alignment and skeletal muscle mechanical properties according to cushion use and backpack load. If a detachable cushion provides effects similar to those of an integrated lumbar support backpack, it may offer a practical and economical alternative that can be used with a wide range of backpacks.

## 2. Materials and Methods

### 2.1. Participants

The sample size was calculated using the G-power software (version 3.1.9.7; Heinrich Heine University, Dusseldorf, Germany). For effect-size estimation, we referred to the study by In et al. [7], which investigated changes in sagittal spinal alignment in response to lumbar support during backpack use. Among the outcomes reported by In et al., the thoracic kyphosis angle was selected as a biomechanically relevant reference outcome because of its relationship with upper-trunk alignment and the UT region. Based on the effect size reported in that study (*η*^2^*p* = 0.05), the corresponding Cohen’s *f* was calculated as 0.23. Based on an alpha level of 0.05 and statistical power of 0.95, the required sample size was estimated to be 27 participants. A total of 29 participants were initially recruited to account for potential exclusions or attrition. One participant did not meet the eligibility criteria; thus, the final sample size was 28.

Healthy men aged 19–29 years were recruited for the study. Individuals with neurological or musculoskeletal disorders or activity limitations caused by neck or low back pain were excluded. Individuals with forward head posture, defined as a craniovertebral angle (CVA) of less than 50°, were also excluded [8,9,10]. Twenty-nine individuals were screened, and one did not meet the eligibility criteria, leaving 28 participants in the final sample. The participant characteristics are presented in Table 1.

This study was approved by the Institutional Review Board of Gachon University (approval No. 1044396-202604-HR-105-01; approved 28 May 2026) and registered with the Clinical Research Information Service (registration No. KCT0012211). All participants received an explanation of the study and provided written informed consent prior to participation.

### 2.2. Experimental Protocol

This single-group repeated-measures study included two within-subject factors: backpack load and lumbar support. The backpack loads were set at baseline and 10%, 15%, and 20% of each participant’s body weight. The lumbar support conditions consisted of two levels: without a lumbar support cushion (WoL) and with a lumbar support cushion (WL).

A commercially available backpack with a 25-L capacity and dimensions of 27.5 cm × 46 cm × 20 cm (Skoll-25, Black Yak, Seoul, Republic of Korea) was used. The backpack was equipped with an integrated waist strap, which was used consistently in both the WL and WoL conditions. The waist strap was used to maintain close contact between the backpack and trunk and to prevent the lower portion of the backpack from moving away from the body. It was not tightened to a degree that produced noticeable compression or additional support around the waist.

The custom-made lumbar support cushion was cylindrical, filled with cotton, and measured 25 cm and 9 cm in length and diameter, respectively. In the WL condition, the cushion was positioned over the L2–L4 region and secured using a separate fixation strap, which was not used in the WoL condition. The fixation strap was made of a nonelastic material similar to that used in a conventional backpack strap and was adjusted only to the extent necessary to maintain the cushion in position over the lumbar region. It was not intentionally tightened to compress the trunk or provide additional lumbar support, thereby minimizing the potential mechanical contribution of the fixation strap.

The position of the backpack was standardized across the participants and experimental conditions. Specifically, the first sacral vertebral (S1) level was identified as an anatomical reference, and the lengths of the bilateral shoulder straps were individually adjusted according to each participant’s body size such that the lower edge of the backpack was positioned at the S1 level [11]. Before measurement under each experimental condition, the backpack position was checked to ensure that its lower edge remained at the S1 level.

After age, height, and body weight were recorded, the backpack loads were individually confirmed to weigh 10%, 15%, and 20% of each participant’s body weight using books. To minimize differences in the center of mass of the load across conditions, books were consistently arranged within the backpack and secured to prevent displacement during measurement.

**Figure 1:**
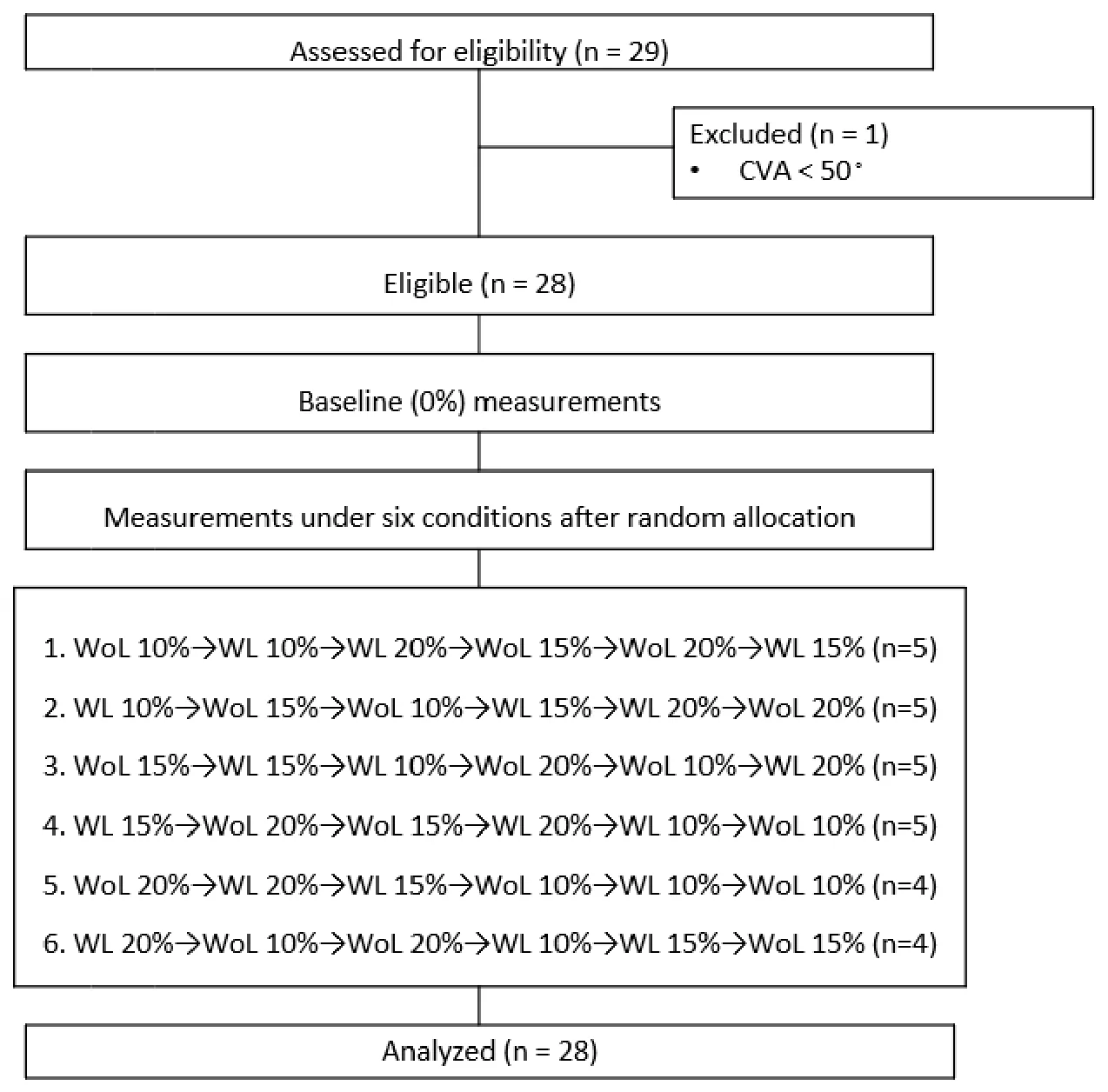
Flow diagram of participant enrollment and study protocol. The arrows represent the sequential flow of the study design.

Including the baseline assessment, each participant completed eight experimental conditions (four load levels and two cushion conditions). The order of the experimental conditions was randomized to achieve counterbalance (Figure 1). Postural angles and skeletal muscle mechanical properties were measured under each condition, with a 5-min rest period between conditions. This interval was selected with reference to previous research to minimize the potential residual effects of transient postural and muscle mechanical changes from the preceding condition and to reduce fatigue associated with repeated measurements [12]. All experimental procedures and measurements were conducted at the Physical Therapy Laboratory of Gachon University, Incheon, Republic of Korea. The entire experimental session was completed in approximately 1 h for each participant.

### 2.3. Measurements

The postural alignment variables included CVA, forward trunk lean (FTL), and pelvic tilt angle (PTA). Muscle variables included tone and stiffness of the UT and SCM.

### 2.4. Assessment of Postural Angles

**Figure 2:**
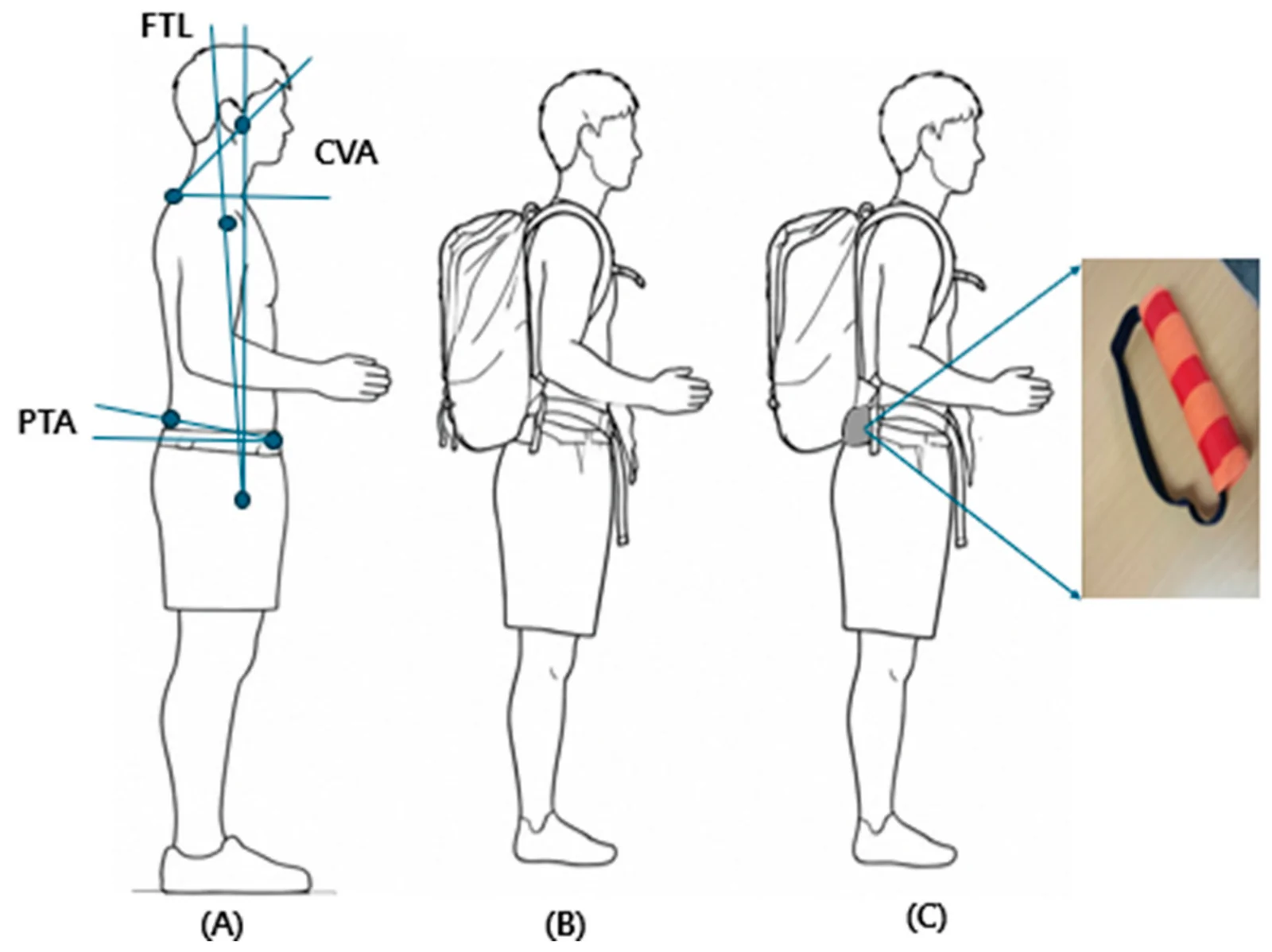
Experimental setup and measurement parameters. (**A**) Biomechanical markers (points) for postural angles: forward trunk lean (FTL), craniovertebral angle (CVA), and pelvic tilt angle (PTA). (**B**) Backpack condition without lumbar support (WoL). (**C**) Backpack condition with lumbar support (WL) and the detachable lumbar support cushion used in the study.

After the backpack was applied under each condition, the participants performed five marching steps in place at a standardized location marked according to their foot position. They then maintained a natural standing posture for 1 min to allow postural sway to stabilize, after which a full-body lateral photograph was obtained from the dominant side (Figure 2). Photogrammetric assessments have demonstrated high inter- and intra-rater reliability in the evaluation of postural alignment [13,14].

Reflective markers were placed over the tragus, spinous process of the seventh cervical vertebra (C7), acromion, anterior superior iliac spine, posterior superior iliac spine, and greater trochanter on the dominant side. Lateral images were obtained using a digital camera (Sony Alpha; Sony, Tokyo, Japan) positioned 3 m away from each participant. The camera was mounted on a tripod, and its height was adjusted to 50% of each participant’s height to minimize measurement errors. A spirit level was used to ensure that the camera lens remained horizontal to the ground, and a constant camera-to-participant distance was maintained across all experimental conditions. Two lateral photographs were obtained for each condition and analyzed using CorelDRAW X7 (Corel Corp., Ottawa, ON, Canada). The CVA, FTL, and PTA were determined from each photograph, and the mean of the two measurements was used as the final value for each condition.

The CVA was defined as the angle between a horizontal line through C7 and the line connecting C7 to the tragus; a smaller value indicated a more pronounced forward head posture. The FTL was defined as the angle between a vertical line through the greater trochanter and the line connecting the greater trochanter to the acromion; a larger value indicated a greater forward trunk inclination. The PTA was defined as the angle between a horizontal line through the anterior superior iliac spine and the line connecting the anterior and posterior superior iliac spines; a larger value indicated greater anterior pelvic tilt [8,15,16].

### 2.5. Assessment of Skeletal Muscle Mechanical Properties

Immediately after lateral photography, the UT and SCM tone and stiffness were measured in the same standing posture using a handheld myotonometer (Myoton PRO; Myoton AS, Tallinn, Estonia). The UT measurement site was defined as the midpoint of the line connecting the acromion and the C7 spinous process, whereas the SCM measurement site was defined as the midpoint between its anterior attachment on the manubrium and the mastoid process [16,17,18]. To ensure consistency in measurement locations and procedures across experimental conditions, each measurement site was marked on the skin at the initial measurement and maintained throughout the experiment; the same investigator placed all anatomical markers and performed all Myoton measurements for all participants.

During measurement, the participants maintained a natural and stable standing posture, and the Myoton probe was positioned perpendicular to the skin surface over the target muscle. After contact with the skin, a pre-pressure of 0.18 N was applied, followed by a brief mechanical impulse of 0.4 N, corresponding to a total applied force of 0.58 N. The device recorded the damped oscillations of the muscle tissue induced by the mechanical impulse and derived the corresponding mechanical properties. In the present study, muscle tone (Hz) and stiffness (N/m) were assessed. Each measurement site was assessed twice under each experimental condition, and the mean of the two measurements was finally analyzed. Myoton-based assessment of muscle mechanical properties has demonstrated high test–retest reliability, with intraclass correlation coefficients ranging from 0.79 to 0.96 [19].

### 2.6. Statistical Analysis

The researcher responsible for data analysis was blinded to the experimental conditions because the data were coded such that the condition assignments were not identifiable during the analysis. Statistical analyses were performed using jamovi version 2.7.38 (The Jamovi Project, 2026). A 4 × 2 two-factor repeated-measures analysis of variance (ANOVA) was performed to examine the main effects of backpack load and cushion condition as well as the effects of their interaction on postural angles and skeletal muscle mechanical properties. The backpack load comprised four levels (baseline, 10%, 15%, and 20% of the body weight), and the cushion condition comprised two levels (WL and WoL). For each outcome variable, the mean of two repeated measurements was used for the statistical analysis. The ICC values for the two repeated measurements ranged from 0.743 to 1.000 across the outcome measures and experimental conditions.

The normality of the data was assessed using the Shapiro–Wilk test, and all data satisfied the assumption of normality (*p* > 0.05). The sphericity was assessed using Mauchly’s test. When the assumption of sphericity was violated, degrees of freedom were adjusted according to the ε-value. Specifically, the Huynh–Feldt correction was applied when ε > 0.75, whereas the Greenhouse–Geisser correction was applied when ε ≤ 0.75.

The main effects of the load and cushion conditions as well as the load × cushion condition interaction were examined for each outcome variable. When significant main or interaction effects were identified, subsequent pairwise comparisons were performed using Tukey’s honest significant difference (HSD) test. The mean differences (MDs) between the conditions were calculated for pairwise comparisons. Statistical significance was set at *p* < 0.05 for all analyses.

An additional post hoc exploratory analysis was conducted to examine the potential load × lumbar support condition interactions within the 10–15% load range. This load range was selected because post hoc comparisons of the main effect of the load showed no further significant changes between the 15% and 20% load conditions. For this exploratory analysis, a 2 × 2 repeated-measures ANOVA was performed for each outcome variable with backpack load (10% and 15% of body weight) and lumbar support conditions (WL and WoL). The load × lumbar support condition interaction was the primary focus of this exploratory analysis, and subsequent pairwise comparisons were performed for outcomes that showed an interaction effect. Because these analyses were exploratory, no correction for multiple testing across outcome variables was applied; therefore, the interaction *p*-values are reported as uncorrected *p*-values and interpreted cautiously. Subsequent pairwise comparisons were adjusted using Tukey’s HSD test. The results of the post hoc exploratory analyses showing significant interaction effects (uncorrected *p* < 0.05) are presented in Table S1.

## 3. Results

### 3.1. Postural Angles

A significant main effect of load on CVA was observed (*F* = 27.554, *p* < 0.001, *η*^2^*p* = 0.505). Post hoc comparisons showed that CVA was significantly lower at 10%, 15%, and 20% of the body weight than at baseline (all *p* ≤ 0.003) and at 15% and 20% than at 10% (*p* = 0.003 and *p* < 0.001, respectively). No significant difference was observed between the 15% and 20% conditions (*p* = 0.515). Neither the cushion condition nor the load × cushion interaction had a significant effect (Table 2).

Load had a significant main effect on FTL (*F* = 53.858, *p* < 0.001, *η*^2^*p* = 0.666) (Table 3). Post hoc comparisons showed that the FTL was significantly greater at 10%, 15%, and 20% of the body weight than at baseline (all *p* < 0.001) and at 15% and 20% than at 10% (both *p* < 0.001). No significant difference was observed between the 15% and 20% conditions (*p* = 0.381). Neither the main effect of the cushion condition nor the load × cushion interaction was significant across the full load range. However, the post hoc exploratory analysis restricted to the 10–15% load range showed a load × cushion interaction (*F* = 5.346, uncorrected *p* = 0.029). Within the WL condition, the FTL was significantly greater at 15% than at 10% (MD = −1.813°, *p* < 0.001), whereas the corresponding difference in the WoL condition did not reach the nominal significance threshold (MD = −0.901°, *p* = 0.066) (Table S1).

For PTA, significant main effects of load (*F* = 9.969, *p* < 0.001, *η*^2^*p* = 0.270) and cushion condition (*F* = 91.384, *p* < 0.001, *η*^2^*p* = 0.772), as well as a significant load × cushion interaction (*F* = 20.213, *p* < 0.001, *η*^2^*p* = 0.428), were observed (Table 4 and Table 5). Post hoc comparisons for the main effect of load showed that PTA was significantly greater at 15% and 20% of the body weight than at baseline (*p* = 0.001 and *p* = 0.004, respectively) and at 15% than at 10% (*p* = 0.034). No significant differences were observed between baseline and 10%, between 10% and 20%, or between 15% and 20% (Table 4).

Analysis of the load × cushion interaction showed that, in the WoL condition, PTA did not differ significantly from baseline at 10%, 15%, or 20% of the body weight. In contrast, in the WL condition, the PTA was significantly greater at 10% (*p* = 0.010), 15% (*p* < 0.001), and 20% (*p* < 0.001) than at baseline. PTA was also significantly greater in the WL condition than in the WoL condition at 10%, 15%, and 20%, with between-condition differences of 1.814°, 3.179°, and 3.399°, respectively (all *p* < 0.001) (Table 5).

### 3.2. Skeletal Muscle Mechanical Properties

Load exerted a significant main effect on UT tone (*F* = 43.326, *p* < 0.001, *η*^2^*p* = 0.616) (Table 6). Post hoc comparisons showed that UT tone was significantly greater at 10%, 15%, and 20% of the body weight than at baseline (all *p* < 0.001) and at 15% and 20% than at 10% (*p* = 0.004 and *p* < 0.001, respectively). No significant difference was observed between the 15% and 20% conditions (*p* = 0.347). Neither the main effect of the cushion condition nor the load × cushion interaction was significant.

Load had a significant main effect on UT stiffness also (*F* = 31.925, *p* < 0.001, *η*^2^*p* = 0.551) (Table 6). Post hoc comparisons showed that UT stiffness was significantly greater at 10%, 15%, and 20% than at baseline (all *p* < 0.001) and at 15% and 20% than at 10% (*p* = 0.004 and *p* < 0.001, respectively). No significant difference was observed between the 15% and 20% conditions (*p* = 0.160). Neither the main effect of the cushion condition nor the load × cushion interaction was significant across the full load range.

However, the post hoc exploratory analysis restricted to the 10–15% load range showed a load × cushion interaction (*F* = 4.366, uncorrected *p* = 0.047). Pairwise comparisons showed that UT stiffness was significantly greater at 15% than at 10% in the WoL condition (MD = −38.76 N/m, *p* = 0.002), whereas the corresponding difference in the WL condition was not significant (MD = −19.37 N/m, *p* = 0.139). UT stiffness at 15% WL was also significantly greater than that at 10% WoL (MD = −33.35 N/m, *p* = 0.004). Given the exploratory nature of this analysis and the use of an uncorrected interaction *p*-value, these findings should be interpreted cautiously (Table S2).

Load (*F* = 12.245, *p* < 0.001, *η*^2^*p* = 0.320) and cushion condition (F = 5.273, *p* = 0.030, *η*^2^*p* = 0.169) had significant main effects on SCM tone (Table 7). Post hoc comparisons showed that the SCM tone was significantly greater at 10%, 15%, and 20% of the body weight than at baseline (*p* = 0.013, *p* = 0.003, and *p* < 0.001, respectively), whereas no significant differences were observed among the three load conditions. The SCM tone was 0.111 Hz greater under the WoL condition than under the WL condition (*p* = 0.030). The load × cushion interaction was not significant.

Load had a significant main effect on SCM stiffness (*F* = 4.272, *p* = 0.027, *η*^2^*p* = 0.137) (Table 7). Post hoc comparisons showed that SCM stiffness was significantly greater at 15% than at baseline (MD = −8.096 N/m, *p* = 0.019), whereas no other pairwise comparisons were significant. Neither the main effect of the cushion condition nor the load × cushion interaction was significant.

## 4. Discussion

This repeated-measures study evaluated changes in postural alignment and mechanical properties of the neck and shoulder muscles according to backpack loads equivalent to 10%, 15%, and 20% of the body weight and the use of a detachable lumbar support cushion. The principal findings were as follows: First, most variables changed significantly with increasing load, and these changes were most pronounced at up to 15% of body weight. Second, cushion use had no significant main effect on the CVA or FTL but increased the PTA. Third, SCM tone was lower under the WL condition.

The CVA decreased, and the FTL increased as the backpack load increased. Because the center of mass of a loaded backpack is positioned posterior to the body, balance may be maintained by leaning the trunk forward and positioning the head anteriorly. These findings are consistent with previous reports of significant changes in CVA and FTL at loads equivalent to 10% or 15% of the body weight [4,20]. In the present study, no additional angular changes were observed between 15% and 20%. This plateau may indicate that primary postural compensation had already been established at 15% or that further increases in load were accommodated through other mechanisms, such as lower-limb joint strategies or increased muscle activity.

The load and cushion conditions had significant main effects on PTA, as well as a significant interaction. Changes in PTA with increasing load were limited in the WoL condition, whereas the PTA in the WL condition was greater than the baseline at each load level. This finding is consistent with those of previous studies on lumbar support backpacks and suggests that the lumbar support cushion promotes an increase in anterior pelvic tilt [7]. However, whether this reflects a corresponding change in lumbar lordosis remains hypothetical, as lumbar curvature was not directly measured. In contrast, changes in PTA with increasing backpack load were relatively limited in the WoL condition compared to those in the WL condition. This may be because, in the absence of lumbar support, postural responses to increasing backpack load may have occurred primarily through changes in CVA and FTL rather than through pelvic adjustment. Consistent with this interpretation, the present study demonstrated that the CVA changes more sensitively with increasing load in the WoL condition than in the WL condition. Previous studies also reported significant increases in cervical lordosis and thoracic kyphosis during backpack carriage [21].

From a clinical perspective, an increase in the anterior pelvic tilt may have different implications depending on an individual’s baseline postural alignment. Appropriate anterior lumbar support may be beneficial for individuals with reduced lumbar lordosis, such as those with a flat-back posture. However, excessive anterior support may increase lumbar lordosis or cause discomfort in individuals who already exhibit pronounced anterior pelvic tilt. Therefore, future studies should consider the baseline postural characteristics and identify subgroups that may respond differently to lumbar support. It should be noted that the baseline lumbar curvature was not directly measured or used to classify participants in the present study; therefore, this clinical application remains speculative and requires confirmation in populations with a confirmed flat-back posture.

UT tone and stiffness increased significantly with increasing backpack load. These changes may reflect the combined effects of the direct mechanical pressure from the shoulder straps, passive tension in the tissues surrounding the shoulder girdle, and increased muscular activity required to maintain postural stability under greater loads. These findings are consistent with those of previous studies reporting increased trapezius muscle activity and fatigue during prolonged backpack carriage as well as increased UT tone and stiffness associated with a forward head posture [22,23].

In the post hoc exploratory analysis, an interaction effect of the lumbar support cushion was observed only within the specific load range of 10–15% of the body weight, with the increase in UT stiffness being more pronounced in the WoL condition (Table S2). In the WoL condition, increased FTL and decreased CVA were observed without substantial changes in pelvic position. This pattern indicates that load-related postural changes occurred primarily in the upper body rather than in the pelvis. These findings suggest that the postural changes associated with the increased PTA observed with the lumbar support cushion may influence changes in UT stiffness. However, because this finding was derived from a post hoc exploratory analysis limited to 10–15% load, it should be interpreted with caution and requires further investigation to clarify this relationship and its potential underlying mechanisms.

SCM tone was greater under all load conditions than at baseline and was greater in the WoL condition than in the WL condition. The SCM is a principal muscle involved in controlling the head and neck position [24], and both deep and superficial neck muscles respond sensitively to vestibular input [25]. Thus, the increase in SCM tone may represent a neuromuscular response associated with controlling the anterior displacement of the head rather than a direct effect of mechanical compression from the backpack load. This pattern suggests that the increased PTA observed with the lumbar support cushion may be associated with lower SCM tone.

However, no significant changes in SCM stiffness were observed in the WL condition. Stiffness represents the resistance of tissue to deformation in response to an external force or muscle contraction. This finding may be explained by the anatomical and mechanical differences between the SCM and UT. Unlike the UT, which is directly subjected to mechanical compression from backpack shoulder straps, the SCM is not located in an area where the backpack load is directly transmitted. Thus, changes in SCM tone may primarily reflect a neuromuscular response associated with controlling and stabilizing the position of the head rather than a direct mechanical response to the backpack load. By contrast, because the SCM is not directly exposed to external compression from the backpack, the mechanical stimulus may have been insufficient to produce a significant change in SCM stiffness.

This study has several limitations. First, the participants were young adult men. Therefore, the findings may not be generalizable to women, older adults, or individuals with musculoskeletal disorders. Second, only short-term responses during static standing were assessed, and dynamic changes during walking, stair negotiation, and prolonged carriage were not evaluated. Although a 5-min rest interval was provided between the experimental conditions to minimize potential carry-over effects, whether this interval was sufficient to completely eliminate residual effects from the preceding condition was not specifically established. Third, lumbar lordosis, thoracic kyphosis, shoulder-strap pressure, scapular kinematics, and muscle activity were not directly measured, precluding confirmation of the mechanisms underlying the observed effects of the lumbar support cushion. Fourth, several outcome variables and additional interval-specific analyses were examined. Therefore, the potential influence of multiple comparisons and the exploratory nature of these analyses should be carefully considered when interpreting the findings. Future studies should include more diverse populations, dynamic tasks, prolonged backpack carriage, and direct assessments of spinal alignment, muscle activity, and load distribution.

## 5. Conclusions

This study investigated the effects of a detachable backpack lumbar support cushion on postural alignment and the mechanical properties of the neck and shoulder muscles across different backpack loads. The use of the lumbar support cushion was associated with greater PTA. In the exploratory analysis of the 10% and 15% body weight conditions, a greater increase in UT stiffness was observed in the WoL condition than in the WL condition. SCM tone was also generally higher in the WoL condition than in the WL condition. These findings suggest that the lumbar support cushion may influence the postural responses and the mechanical properties of the neck and shoulder muscles during backpack carriage. One possible explanation is that changes in the PTA associated with lumbar support may alter compensatory postural responses in the upper body; however, this mechanism remains hypothetical because thoracic kyphosis and muscle activity were not directly measured in this study. Overall, a detachable lumbar support cushion may provide a practical means for modifying postural responses during backpack carriage.

## Figures and Tables

**Table 1:** General characteristics of the participants (mean ± standard deviation).

Variable	Value (*n* = 28)
Age (years)	22.96 ± 3.33
Height (cm)	174.96 ± 5.96
Weight (kg)	73.84 ± 12.47
Body mass index (kg/m^2^)	24.01 ± 3.24

**Table 2:** Two-way repeated-measures ANOVA and Tukey post hoc comparisons for CVA.

Repeated-Measures Comparison				Post Hoc Comparison (Tukey)					
Factors	F	*p*	*η* ^2^ *p*	Comparison		MD (°)	SE	*t*	*p* (Tukey)
Load	27.554	<0.001 *	0.505	Base	10	1.911	0.440	4.338	<0.001 *
					15	2.751	0.422	6.513	<0.001 *
					20	3.073	0.537	5.723	<0.001 *
				10	15	0.840	0.216	3.896	0.003 *
					20	1.162	0.260	4.473	<0.001 *
				15	20	0.322	0.231	1.392	0.515
Cushion	0.680	0.417	0.025	WoL	WL	0.092	0.112	0.825	0.417

Abbreviations: ANOVA, analysis of variance; CVA, craniovertebral angle; MD, mean difference; SE, standard error; WL, with lumbar support cushion; WoL, without lumbar support cushion. * indicates a statistically significant difference (*p* < 0.05).

**Table 3:** Two-way repeated-measures ANOVA and Tukey post hoc comparisons for FTL.

Repeated-Measures Comparison				Post Hoc Comparison (Tukey)					
Factors	*F*	*p*	*η* ^2^ *p*	Comparison		MD (°)	SE	*t*	*p* (Tukey)
Load	53.858	<0.001 *	0.666	Base	10	−3.271	0.466	−7.025	<0.001 *
					15	−4.627	0.602	−7.682	<0.001 *
					20	−5.097	0.624	−8.166	<0.001 *
				10	15	−1.357	0.233	−5.816	<0.001 *
					20	−1.826	0.268	−6.803	<0.001 *
				15	20	−0.469	0.288	−1.627	0.381
Cushion	0.363	0.552	0.013	WoL	WL	−0.068	0.113	−0.602	0.552

Abbreviations: ANOVA, analysis of variance; FTL, forward trunk lean; MD, mean difference; SE, standard error; WL, with lumbar support cushion; WoL, without lumbar support cushion. * indicates a statistically significant difference (*p* < 0.05).

**Table 4:** Two-way repeated-measures ANOVA and Tukey post hoc comparisons for PTA.

Repeated-Measures Comparison				Post Hoc Comparison (Tukey)					
Factors	*F*	*p*	*η* ^2^ *p*	Comparison		MD (°)	SE	*t*	*p* (Tukey)
Load	9.969	<0.001 *	0.270	Base	10	−0.980	0.432	−2.269	0.131
					15	−2.228	0.518	−4.304	0.001 *
					20	−2.062	0.537	−3.837	0.004 *
				10	15	−1.248	0.429	−2.906	0.034 *
					20	−1.082	0.493	−2.196	0.150
				15	20	0.166	0.356	0.467	0.966
Cushion	91.384	<0.001 *	0.772	WoL	WL	−2.097	0.219	−9.559	<0.001 *

Abbreviations: ANOVA, analysis of variance; PTA, pelvic tilt angle; MD, mean difference; SE, standard error; WL, with lumbar support cushion; WoL, without lumbar support cushion. * indicates a statistically significant difference (*p* < 0.05).

**Table 5:** Interaction effects and post hoc analysis of PTA.

Repeated-Measures Comparison				Post Hoc Comparison (Tukey)							
Factors	*F*	*p*	*η* ^2^ *p*	Load (1)	Cushion (1)	Load (2)	Cushion (2)	MD (°)	SE	*t*	*p* (Tukey)
Load × Cushion	20.213	<0.001 *	0.428	Base	WoL	Base	WL	0.005	0.025	0.185	1.000
						10	WoL	−0.071	0.454	−0.155	1.000
							WL	−1.885	0.477	−3.955	0.010 *
						15	WoL	−0.636	0.520	−1.223	0.918
							WL	−3.816	0.598	−6.378	<0.001 *
						20	WoL	−0.360	0.555	−0.649	0.998
							WL	−3.759	0.629	−5.973	<0.001 *
					WL	10	WoL	−0.075	0.454	−0.166	1.000
							WL	−1.890	0.479	−3.949	0.010 *
						15	WoL	−0.641	0.518	−1.237	0.913
							WL	−3.820	0.597	−6.400	<0.001 *
						20	WoL	−0.365	0.556	−0.655	0.998
							WL	−3.764	0.629	−5.985	<0.001 *
				10	WoL	10	WL	−1.814	0.349	−5.204	<0.001 *
						15	WoL	−0.566	0.350	−1.619	0.735
							WL	−3.745	0.608	−6.159	<0.001 *
						20	WoL	−0.289	0.510	−0.567	0.999
							WL	−3.689	0.632	−5.833	<0.001 *
					WL	15	WoL	1.249	0.442	2.824	0.131
							WL	−1.931	0.594	−3.249	0.054
						20	WoL	1.525	0.514	2.968	0.098
							WL	−1.874	0.649	−2.887	0.115
				15	WoL	15	WL	−3.179	0.425	−7.473	<0.001 *
						20	WoL	0.276	0.434	0.637	0.998
							WL	−3.123	0.478	−6.537	<0.001 *
					WL	20	WoL	3.456	0.560	6.172	<0.001 *
							WL	0.056	0.461	0.122	1.000
				20	WoL	20	WL	−3.399	0.504	−6.750	<0.001 *

Abbreviations: PTA, pelvic tilt angle; MD, mean difference; SE, standard error; WL, with lumbar support cushion; WoL, without lumbar support cushion. * indicates a statistically significant difference (*p* < 0.05).

**Table 6:** Two-way repeated-measures ANOVA and Tukey post hoc comparisons for UT mechanical properties.

	Repeated-Measures Comparison				Post Hoc Comparison (Tukey)					
	Factors	*F*	*p*	*η* ^2^ *p*	Comparison		MD	SE	*t*	*p* (Tukey)
Tone (Hz)	Load	43.326	<0.001 *	0.616	Base	10	−1.621	0.284	−5.715	<0.001 *
						15	−2.706	0.364	−7.436	<0.001 *
						20	−3.082	0.349	−8.833	<0.001 *
					10	15	−1.085	0.286	−3.794	0.004 *
						20	−1.462	0.250	−5.835	<0.001 *
					15	20	−0.377	0.223	−1.693	0.347
	Cushion	0.267	0.610	0.010	WoL	WL	−0.065	0.126	−0.516	0.610
Stiffness (N/m)	Load	31.925	<0.001 *	0.551	Base	10	−38.493	7.104	−5.419	<0.001 *
						15	−67.558	12.171	−5.551	<0.001 *
						20	−82.669	11.702	−7.065	<0.001 *
					10	15	−29.065	7.645	−3.802	0.004 *
						20	−44.176	7.444	−5.934	<0.001 *
					15	20	−15.111	6.987	−2.163	0.160
	Cushion	1.159	0.292	0.043	WoL	WL	−3.432	3.187	−1.077	0.292

Abbreviations: ANOVA, analysis of variance; MD, mean difference; SE, standard error; UT, upper trapezius; WL, with lumbar support cushion; WoL, without lumbar support cushion. * indicates a statistically significant difference (*p* < 0.05).

**Table 7:** Two-way repeated-measures ANOVA and Tukey post hoc comparisons for SCM muscle properties.

	Repeated-Measures Comparison				Post Hoc Comparison (Tukey)					
	Factors	*F*	*p*	*η* ^2^ *p*	Comparison		MD	SE	*t*	*p* (Tukey)
Tone (Hz)	Load	12.245	<0.001 *	0.320	Base	10	−0.346	0.104	−3.332	0.013 *
						15	−0.454	0.114	−3.991	0.003 *
						20	−0.502	0.111	−4.532	<0.001 *
					10	15	−0.108	0.056	−1.922	0.244
						20	−0.156	0.069	−2.267	0.132
					15	20	−0.047	0.080	−0.589	0.935
	Cushion	5.273	0.030 *	0.169	WoL	WL	0.111	0.048	2.296	0.030 *
Stiffness (N/m)	Load	4.272	0.027 *	0.137	Base	10	−5.364	2.470	−2.172	0.157
						15	−8.096	2.569	−3.151	0.019 *
						20	−7.757	3.835	−2.023	0.205
					10	15	−2.732	1.690	−1.617	0.386
						20	−2.393	2.371	−1.009	0.745
					15	20	0.339	1.843	0.184	0.998
	Cushion	0.167	0.686	0.006	WoL	WL	0.398	0.975	0.408	0.686

Abbreviations: ANOVA, analysis of variance; MD, mean difference; SCM, sternocleidomastoid; SE, standard error; WL, with lumbar support cushion; WoL, without lumbar support cushion. * indicates a statistically significant difference (*p* < 0.05).

## Data Availability

The data presented in this study are available upon request from the corresponding author.

## Supplementary Materials

The following supporting information can be downloaded at https://www.mdpi.com/article/10.3390/jcm15207900/s1, Table S1: Interaction Effects and Post-hoc Analysis of FTL at 10% and 15% Load Conditions. Table S2: Interaction Effects and Post-hoc Analysis of UT stiffness at 10% and 15% Load Conditions.

## Abbreviations

The following abbreviations are used in this manuscript:

ANOVA	Analysis of variance
C7	Seventh cervical vertebra
CVA	Craniovertebral angle
FTL	Forward trunk lean
MD	Mean difference
PTA	Pelvic tilt angle
SCM	Sternocleidomastoid
SE	Standard error
UT	Upper trapezius
WL	With lumbar support cushion
WoL	Without lumbar support cushion
HSD	Honest significant difference
