# Supplementary material for: Effects of a Lumbar Support Cushion in a Backpack on Postural Alignment and Skeletal Muscle Mechanical Properties: A Repeated-Measures Trial in Healthy Adults
Source: J Clin Med. 2026 Oct 13;15(20):7900. doi: 10.3390/jcm15207900 (PMC13706140; doi:10.3390/jcm15207900)
Supplement: Supplementary file 1 [file jcm-15-07900-s001.zip › jcm-4527821-supplementary.pdf]

Table S1. Interaction Effects and Post-hoc Analysis of FTL at 10% and 15% Load Conditions.

**Repeated-Measures Comparison**			**Post Hoc Comparison (Tukey)**							
**Factors**	***F***	***Uncor-rected p***	**Load (1)**	**Cush-ion (1)**	**Load (2)**	**Cush ion (2)**	**MD (°)**	**SE**	***t***	***p* (Tukey)**
Load x Cushion	5.346	0.029*	10	WoL	10	WL	0.285	0.223	1.283	0.581
					15	WoL	-0.901	0.345	-2.608	0.066
						WL	-1.528	0.334	-4.578	0.001*
				WL	15	WoL	-1.186	0.224	-5.291	< .001*
						WL	-1.813	0.260	-6.980	< .001*
			15	WoL	15	WL	-0.627	0.285	-2.202	0.148

Abbreviations: FTL, forward trunk lean; MD, mean difference; SE, standard error; WL, with lumbar support cushion; WoL, without lumbar support cushion. * indicates a statistically significant difference *(p* < 0.05).

Table S2. Interaction Effects and Post-hoc Analysis of UT stiffness at 10% and 15% Load Conditions.

**Repeated-Measures Comparison**			**Post Hoc Comparison (Tukey)**							
Factors	*F*	***Uncor-rected p***	**Load (1)**	**Cush ion (1)**	**Load (2)**	**Cush ion (2)**	**MD(N/m)**	**SE**	***t***	***p* (Tukey)**
Load x Cushion	4.366	0.047*	10	WoL	10	WL	-13.98	5.418	-2.581	0.071
					15	WoL	-38.76	9.236	-4.197	0.002*
						WL	-33.35	8.731	-3.820	0.004*
				WL	15	WoL	-24.78	9.219	-2.688	0.056
						WL	-19.37	8.639	-2.242	0.139
			15	WoL	15	WL	5.407	7.618	0.710	0.892

Abbreviations: MD, mean difference; SE, standard error; UT, upper trapezius; WL, with lumbar support cushion; WoL, without lumbar support cushion. * indicates a statistically significant difference (*p* < 0.05).
